# Granzyme B mediates both direct and indirect cleavage of extracellular matrix in skin after chronic low-dose ultraviolet light irradiation

**DOI:** 10.1111/acel.12298

**Published:** 2014-12-11

**Authors:** Leigh G Parkinson, Ana Toro, Hongyan Zhao, Keddie Brown, Scott J Tebbutt, David J Granville

**Affiliations:** 1 Centre for Heart Lung Innovation, St. Paul's HospitalVancouver, British Columbia, Canada; 2 Department of Pathology and Laboratory Medicine, University of British ColumbiaVancouver, British Columbia, Canada; 3 Department of Medicine, Division of Respiratory Medicine, University of British ColumbiaVancouver, British Columbia, Canada

**Keywords:** aging, collagen, extracellular matrix, fibronectin, granzyme B, matrix metalloproteinase, photoaging, skin, ultraviolet light

## Abstract

Extracellular matrix (ECM) degradation is a hallmark of many chronic inflammatory diseases that can lead to a loss of function, aging, and disease progression. Ultraviolet light (UV) irradiation from the sun is widely considered as the major cause of visible human skin aging, causing increased inflammation and enhanced ECM degradation. Granzyme B (GzmB), a serine protease that is expressed by a variety of cells, accumulates in the extracellular milieu during chronic inflammation and cleaves a number of ECM proteins. We hypothesized that GzmB contributes to ECM degradation in the skin after UV irradiation through both direct cleavage of ECM proteins and indirectly through the induction of other proteinases. Wild-type and GzmB-knockout mice were repeatedly exposed to minimal erythemal doses of solar-simulated UV irradiation for 20 weeks. GzmB expression was significantly increased in wild-type treated skin compared to nonirradiated controls, colocalizing to keratinocytes and to an increased mast cell population. GzmB deficiency significantly protected against the formation of wrinkles and the loss of dermal collagen density, which was related to the cleavage of decorin, an abundant proteoglycan involved in collagen fibrillogenesis and integrity. GzmB also cleaved fibronectin, and GzmB-mediated fibronectin fragments increased the expression of collagen-degrading matrix metalloproteinase-1 (MMP-1) in fibroblasts. Collectively, these findings indicate a significant role for GzmB in ECM degradation that may have implications in many age-related chronic inflammatory diseases.

## Introduction

Aging is a complex and time-dependent biological process that affects all organ systems and is characterized by a decline in function and a reduced ability of the body to respond to stress due to physical, biological, and chemical agents. As the average age of the Western population increases, with predictions for individuals aged 60 years or older to account for 26% of the population in the USA by the year 2050 (Administration on Aging, 2010), aging and age-related diseases are becoming a growing issue and problem.

Chronic low-grade inflammation and oxidative stress have been proposed as causative agents of aging and age-related disease (Hendel *et al*., 2010; Chung *et al*., 2011). The skin represents an ideal model organ for aging research because of its accessibility, thus allowing the study of intrinsic and extrinsic/environmental factors contributing to the complex phenomenon of aging, injury, and repair (Wenk *et al*., 2001). Among all the environmental factors, solar UV irradiation is the most influential in premature aging of the skin, causing 80–90% of the morphological, structural, and biochemical changes collectively termed photoaging (Gilchrest & Krutmann, 2006). Chronically irradiated skin is metabolically hyperactive and is characterized by epidermal hyperplasia, reduced/disorganized collagen, and enhanced inflammation (Bosset *et al*., 2003).

Over recent years, substantial progress has been made in elucidating the underlying molecular mechanisms of UV-induced photoaged skin. It is generally accepted that UVB (280–320 nm) and UVA (320–400 nm) irradiation generate severe oxidative stress in skin cells, resulting in transient and permanent genetic damage, increased AP-1 activity, increased MMP expression, impaired TGF-β signaling, enhanced collagen degradation, and decreased collagen synthesis (Pillai *et al*., 2005; Gilchrest & Krutmann, 2006).

Granzyme B (GzmB) is a serine protease that has been traditionally studied in immune cell-mediated apoptosis, once believed to be released exclusively from cytotoxic lymphocytes and natural killer cells, along with the pore-forming protein perforin, to induce apoptotic cell death (Jenne & Tschopp, 1988). However, recent work has focused on extracellular GzmB due to emerging evidence that GzmB accumulates in the extracellular milieu and/or body fluids of many age-related or inflammatory diseases, and retains its activity (Boivin *et al*., 2009). It is now known that GzmB can be released constitutively and nonspecifically to the extracellular space by lymphocytes and NK cells in the absence of target cell engagement and can be induced in many other types of immune (e.g., mast cells, basophils, dendritic cells, B cells) and nonimmune cells (e.g., keratinocytes, chondrocytes) that often do not express perforin or form immunological synapses with target cells (Pardo *et al*., 2007; Boivin *et al*., 2009; Prakash *et al*., 2009). Of particular relevance to the skin and UV irradiation, both UVA and UVB irradiation induce GzmB expression in keratinocytes, both *in vitro* and in human skin (Hernandez-Pigeon *et al*., 2006, 2007). Many extracellular matrix proteins are substrates of GzmB (Buzza *et al*., 2005; Hendel *et al*., 2010; Boivin *et al*., 2012), including decorin and fibronectin, which have important roles in collagen fibrillogenesis and integrity, and cell attachment and signaling, respectively (Danielson *et al*., 1997; Romberger, 1997; Geng *et al*., 2006). Furthermore, a role of GzmB has emerged in dermatological indications with studies implicating extracellular GzmB activity in diet-induced models of skin aging and impaired wound healing (Hiebert *et al*., 2011, 2013; Hsu *et al*., 2014). In these studies, GzmB deficiency protected against a loss in dermal collagen density and skin thinning, and accelerated wound closure.

As greater than 80% of skin aging is believed to be due to UV exposure (Gilchrest & Krutmann, 2006), and GzmB is expressed by many of the cells present or recruited after irradiation, we hypothesized that GzmB contributes to extracellular matrix degradation after UV irradiation through both direct cleavage of ECM proteins and indirectly through the induction of other proteinases, which leads to a phenotype of aged skin. Using a mouse model of chronic, low-grade UV irradiation over 20 weeks, we demonstrated that GzmB deficiency protects against wrinkle formation and a loss in collagen density, while GzmB-mediated fibronectin and decorin cleavage contributed to increased MMP expression and collagen degradation. This work demonstrates an important role for GzmB in aging skin, which may have implications in many other aged-related chronic inflammatory diseases where ECM degradation is a hallmark.

## Results

### GzmB deficiency protects against wrinkle formation

**Figure 1:**
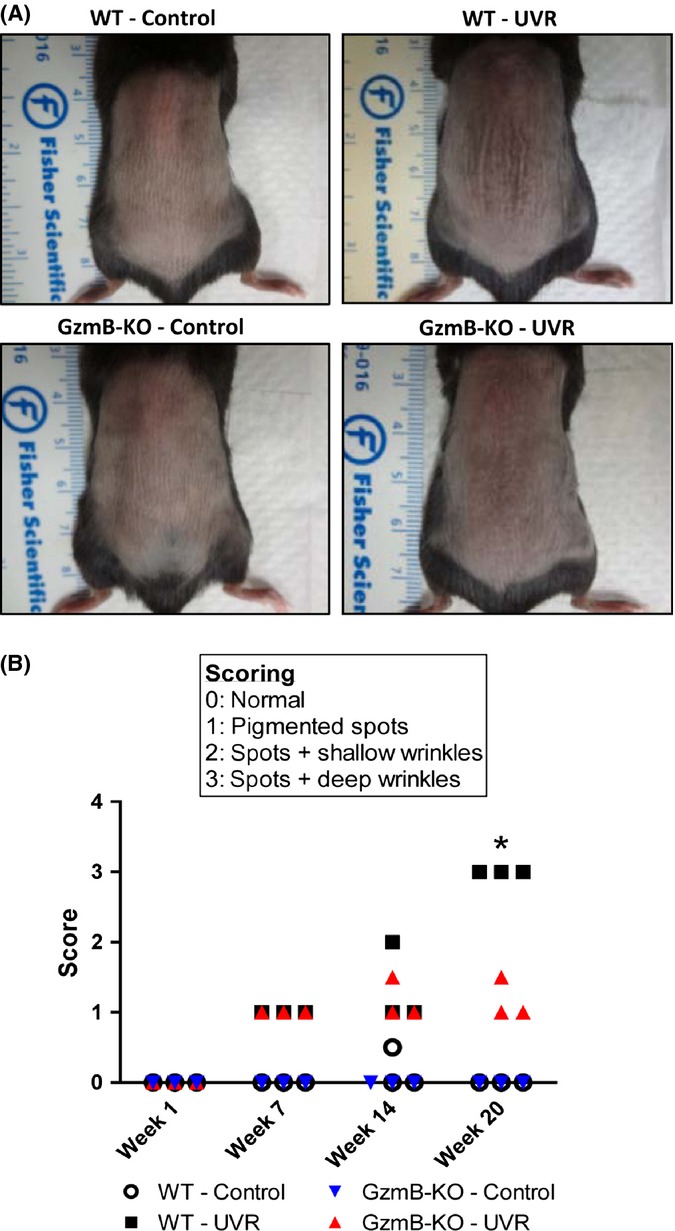
GzmB deficiency protects against wrinkle formation. (A) Representative images of wild-type (WT) and GzmB-knockout mice (KO) exposed to chronic low-grade UV irradiation (UVR) for 20 weeks. Nonirradiated controls were included for comparison. (B) Three independent scorers blinded to time, genotype, and treatment assessed each mouse, and results are presented as median scores for each scorer (*P < 0.05, Wilcoxon signed rank).

Wild-type (WT) and GzmB-knockout (GzmB-KO) mice were repeatedly exposed to solar-simulated UV irradiation (UVR) for 20 weeks. Representative digital images of mice after the 20 week treatment period are shown in Fig.1A. Each mouse in the study was scored on a scale of UV damage by three independent assessors blinded to time, genotype, and treatment (Fig.1B; results expressed at median values for each scorer). Over the course of the 20-week study, WT and GzmB-KO nonirradiated control mice had no visible changes in skin appearance, achieving an expected score of 0. By week 7, both WT and GzmB-KO mice exposed to UV developed dark-pigmented spots over their mid-dorsum. After 20 weeks of UV exposure, there was a significant difference in the appearance of wrinkles between WT and GzmB-KO mice, with GzmB deficiency protecting against the formation of deep, coarse wrinkles (Fig1).

### Increased GzmB expression in UV-irradiated skin

**Figure 2:**
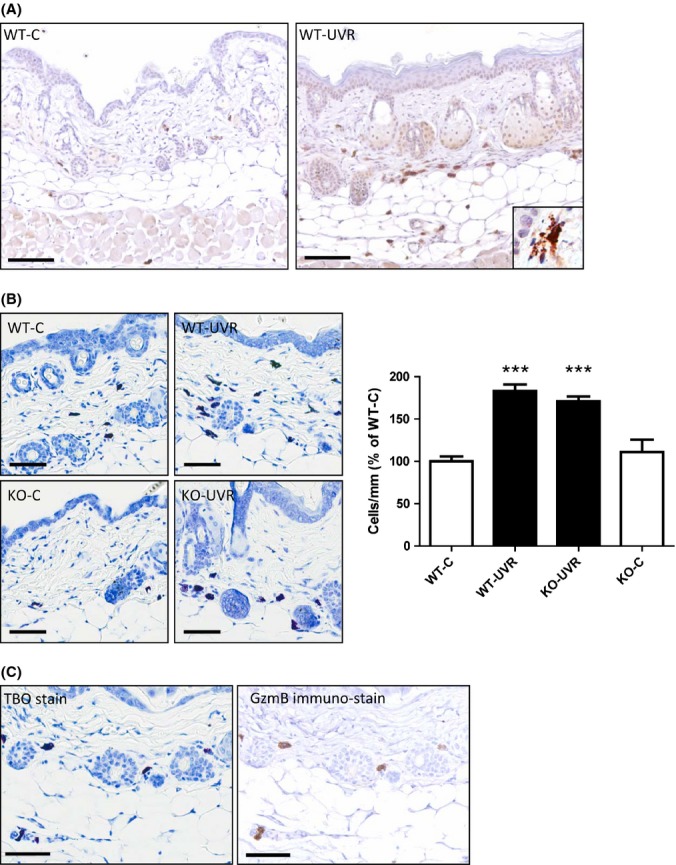
Increased GzmB expression and mast cells in UV-irradiated skin. (A) Dorsal skin sections from nonirradiated (WT-C) and UV-irradiated (WT-UVR) mice immunostained for GzmB. Insert – magnified view of degranulating cell with GzmB immunopositivity. Scale bars = 100 μm. (B) Dorsal skin sections from both nonirradiated (C) and UV-treated (UVR), wild-type (WT), and granzyme B-knockout (KO) mice were stained with TBO, and the number of mast cells was counted (mean ± SEM; ***P < 0.001 Tukey's multiple comparison). Scale bars = 60 μm. (C) Direct colocalization of GzmB immunopositivity with mast cells. Sections were first stained with TBO (pH = 2), and the same sections were then immunostained for GzmB following image capture. Scale bars = 60 μm.

Following 20 weeks UV irradiation, dorsal skin samples were collected and GzmB expression was assessed via immunohistochemistry. Compared to wild-type nonirradiated control animals (WT-C), UV-treated skin (WT-UVR) exhibited a marked increase in GzmB expression with both intracellular and extracellular staining (Fig.2A). Increased staining was observed in the keratinocytes of the epidermis as well as in the epithelial cells of hair follicles. A large cellular infiltrate also stained positively for GzmB. These cells, determined to be mast cells (see next section), had intact and degranulated phenotypes, in which GzmB appeared to be released from the cell (Fig.2A, WT-UVR, insert).

Histological analyses of skin from UV-treated animals showed typical morphological changes associated with chronic UV exposure. There was a significant increase in epidermal and dermal thicknesses in both WT and GzmB-KO mice, leading to an overall increase in skin thickness (Fig. S1).

### GzmB colocalizes to an increased mast cell population

Mast cells were visualized and quantified in mouse skin samples using toluidine blue (TBO) staining (pH = 2). There was a significant increase in the number of mast cells in the skin of both WT and GzmB-KO mice after UV exposure as compared to control mice (WT-C 17.3 ± 1.0; WT-UVR 31.8 ± 1.3; KO-UVR 29.7 ± 1.0; KO-C 19.2 ± 2.5 cells/mm; Fig.2B) The concomitant increase in mast cells in both genotypes along with similar increases in skin thickness indicates that GzmB deficiency did not affect the immune cell recruitment and tissue response after UV exposure and that GzmB deficiency had no adverse affects on these processes. This is supported by the quantification of other immune cells (neutrophils, macrophages, T cells), which showed similar results (Fig. S2).

Many of the GzmB positively stained cells had a mast cell phenotype (Fig.2A). To investigate the colocalization of GzmB to mast cells, skin sections were first stained with TBO for mast cells. Subsequently, sections were immunostained for GzmB. A direct colocalization of GzmB immunopositivity with mast cells in wild-type control and UV-exposed animals was observed (Fig.2C).

### GzmB deficiency protects against loss of dermal collagen density and fibronectin degradation in UV-irradiated skin

**Figure 3:**
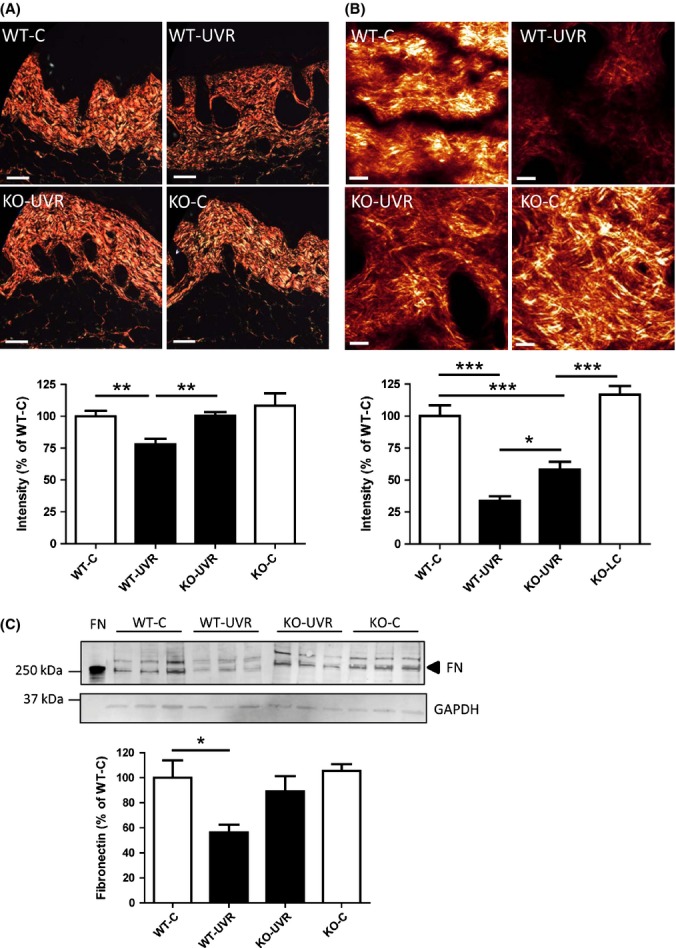
GzmB deficiency protects against loss of dermal collagen density and fibronectin in UV-irradiated skin. (A) Dorsal skin sections were stained with picrosirius red and visualized under polarized light. Quantification of collagen was achieved by color segmentation and intensity analysis of stained sections. Scale bars = 100 μm. (B) Second-harmonic generation signals of collagen from intact *ex vivo* skin samples were quantified as a measure of collagen density. Scale bars = 20 μm (*P < 0.05; **P < 0.01; ***P < 0.001 Tukey's multiple comparison). (C) Fibronectin was assessed in tissue homogenates via Western blot. Results are presented as a percentage of wild-type nonirradiated control (WT-C) (mean ± SEM; *P < 0.05 Dunnett's multiple comparison).

Loss of dermal collagen density and organization is a hallmark of chronically aged skin and contributes to macroscopic changes in skin appearance (Fisher *et al*., 1997). Collagen density in mouse skin samples was examined by picrosirius red staining, and multiphoton microscopy. Picrosirius red staining was observed under polarized light, and dermal collagen from wild-type control (WT-C), GzmB-KO control (KO-C), and GzmB-KO UV-irradiated (KO-UVR) mice exhibited bright red/orange staining indicative of thick, dense collagen fibres at the 20-week time point. In contrast, there was a diminished intensity of staining of dermal collagen in the skin of wild-type UV-irradiated mice (WT-UVR), and collagen bundles appeared to be less tightly packed (Fig.3A). Quantification of the intensity of staining revealed a significant decrease for wild-type UV-irradiated mice compared to all other groups (% of WT-C: WT-UVR 79 ± 4%; KO-UVR 100 ± 3%; KO-C 108 ± 10%; Fig.3A).

Representative second-harmonic generation (SHG) images originating from the collagen matrix are shown in Fig.3B. There was a significant reduction in signal and collagen density from wild-type control to wild-type UV-irradiated mice (34 ± 4% of WT-C), which was significantly protected in GzmB-KO UV-irradiated mice (58 ± 6% of WT-C) (Fig.3B).

GzmB cleavage of human and mouse fibronectin (FN) has been observed in multiple *in vitro* and *in vivo* studies (Buzza *et al*., 2005; Hernandez-Pigeon *et al*., 2007; Hendel & Granville, 2013; Hiebert *et al*., 2013). In this study, UV irradiation induced a significant loss of full-length FN in skin tissue of wild-type mice (56 ± 6% of WT-C; *n* = 6) as compared to controls. However, skin from GzmB-KO mice also exposed to UV had similar levels of FN to controls (95 ± 16% of WT-C; *n* = 6), suggesting there was protection from FN cleavage and degradation in GzmB-deficient mice.

### GzmB causes fibroblast detachment and fibronectin fragmentation *in vitro*

**Figure 4:**
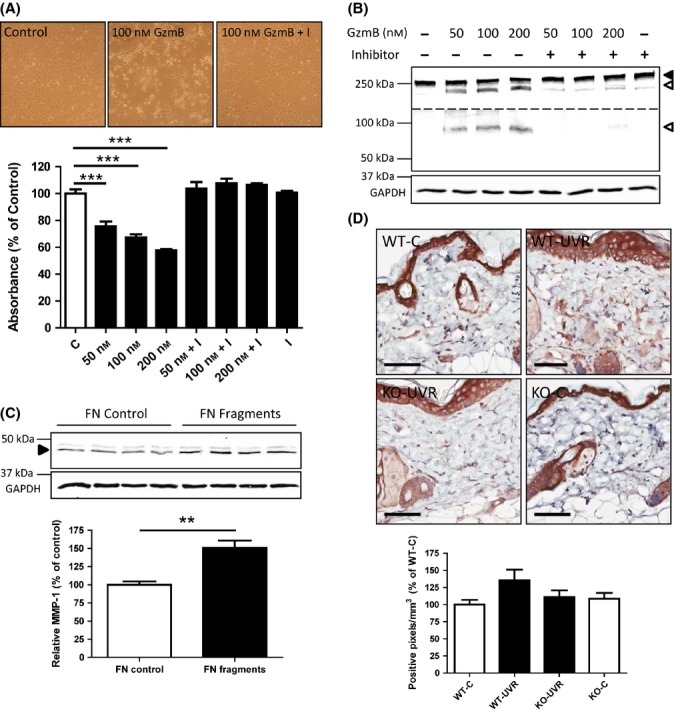
GzmB causes fibroblast detachment and fibronectin fragmentation *in vitro,* and GzmB-mediated FN fragments induce MMP-1 expression in fibroblasts. (A) Mouse fibroblasts plated overnight in complete medium were treated with the indicated concentrations of GzmB ± Inhibitor (I; 50 μm) for 7 h (in serum-free conditions). Adherent cells were assessed by MTS assay after washing once in PBS to remove nonadherent cells (mean ± SEM from quadruplicate wells; ***P < 0.001 Dunnett's multiple comparison). (B) Supernatants collected from (A) were assayed for fibronectin by Western blot. Closed arrowhead = full-length fibronectin; open arrowhead = fibronectin fragments. Although the same blot, the dotted line represents sections presented at different brightness/contrast so that fragments can be clearly observed at different kDa. (C) Primary fibroblasts were added to GzmB-mediated FN fragments, and MMP-1 release was assayed in the supernatants after 20 h by Western blot. GAPDH probed from cell lysates of the same wells served as loading controls. Results are presented as a percentage of intact fibronectin control (mean ± SEM from quadruplicate wells, **P < 0.01 *t*-test). (D) Dorsal skin sections from mice were stained with MMP-1 antibody, and intensity of staining in the dermis (excluding hair follicles) was measured by detecting the number of positive pixels above a set threshold, normalized to area. Results are expressed as a percentage of wild-type control (WT-C) (mean ± SEM). Scale bars = 60 μm.

To rationalize the observations of loss of collagen density *in vivo*, the effect of extracellular GzmB on fibroblast function and FN fragmentation was investigated *in vitro*. In support of previous studies with other cell types (Buzza *et al*., 2005; Pardo *et al*., 2007), recombinant GzmB treatment caused a dramatic change in morphology of 3T3 mouse fibroblasts. Cells rounded up and were observed to detach from culture plates. GzmB treatment resulted in a dose-dependent decrease in the number of adhered cells which was abrogated by cotreatment with Compound 20 (specific GzmB inhibitor) (Fig.4A).

In parallel experiments, supernatants were collected from all wells and aliquot samples were prepared for separation on SDS-PAGE gels and probed for FN by Western blot. GzmB treatment resulted in a dose-dependent increase in FN fragmentation, which was inhibited by cotreatment with the specific GzmB inhibitor, Compound 20 (Fig.4B). These fragments were assayed from the supernatant (treatment in serum-free media) and thus can be assumed to be released from the cell-derived matrix.

### GzmB-mediated FN fragments increase MMP expression in fibroblasts

Primary fibroblasts were added to culture wells coated with FN. Experimental wells coated with FN were first treated with GzmB for 2 h to produce GzmB-mediated FN fragments both in the supernatant and attached to the culture plate (data not shown) (Hendel & Granville, 2013). After inhibition of GzmB with Compound 20, primary fibroblasts were seeded to wells and incubated for 20 h, after which supernatants were collected and assayed for MMP-1 by Western blot. There was a significant increase in the expression and release of MMP-1 from fibroblasts in contact with GzmB-mediated FN fragments compared to controls with intact FN (151 ± 10% of FN control; Fig.4C). Similarly, there was a significant increase in the amount of MMP-3 released to the supernatant (129.1 ± 10% of FN control; Fig. S3).

Skin sections from control and UV-irradiated mice at 20 weeks were immunostained for MMP-1 (Fig.4D). There was intense staining in both the epidermis and hair follicles, which were similar for all control and UV-irradiated mice. However, there was a noticeable difference in the expression of MMP-1 in the dermis, which exhibited both intracellular and extracellular staining. While it failed to reach statistical significance, quantification of the intensity of staining in the dermis (excluding hair follicles) revealed a trend for an increase in the amount of MMP-1 in wild-type UV-irradiated mice, which was not observed in GzmB-KO UV-irradiated mice (Fig.4D).

### GzmB-mediated decorin cleavage renders collagen fibrils more susceptible to degradation by MMP-1

**Figure 5:**
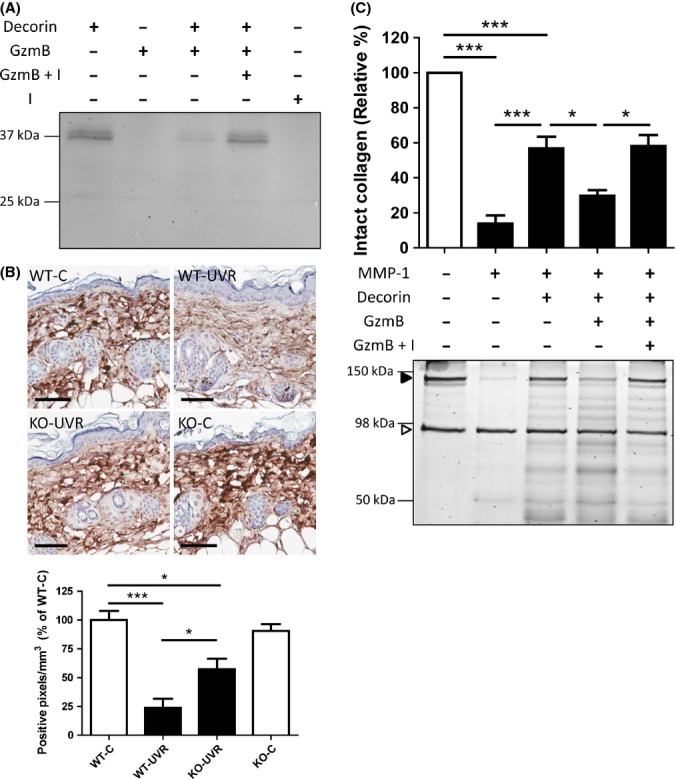
GzmB-mediated decorin cleavage renders collagen fibrils more susceptible to degradation by MMP-1. (A) Decorin cleavage assay. Recombinant decorin (0.4 μg) was treated with GzmB (100 nm) ± Inhibitor (I; 50 μm) for 8 h, 37°C. (B) Dorsal skin sections from control and UV-irradiated mice were stained with decorin antibody. Intensity of staining in the dermis (excluding hair follicles) was measured by detecting the number of positive pixels above a set threshold, normalized to area. Results are expressed as a percentage of wild-type control (WT-C) (mean ± SEM; *P < 0.05; ***P < 0.001 Tukey's multiple comparison). Scale bars = 60 μm. (C) Collagen fibrils ± decorin were treated with or without GzmB (100 nm) ± Compound 20 (I, 50 μm) as indicated. Loss of intact collagen (closed arrowhead) was assessed by SDS-PAGE electrophoresis followed by Coomassie blue staining. Open arrowhead = bovine serum albumin. Results are expressed as a percentage of intact collagen control (mean ± SEM of three independent experiments, *P < 0.05; ***P < 0.001 Tukey's multiple comparison).

Decorin interacts with the surface of collagen fibrils to impede access and proteolytic cleavage by collagenous proteinases (Geng *et al*., 2006). In the present study, GzmB-mediated decorin cleavage rendered collagen fibrils more susceptible to degradation by MMP-1 (Fig.5). GzmB cleaves decorin (Fig.5A). There was a significant decrease in decorin staining in the skin of wild-type UV-irradiated mice (24 ± 8% of WT-C) as compared to wild-type control mice, which was significantly protected in GzmB-KO UV-irradiated mice (57 ± 9% of WT-C) (Fig.5B). Decorin attenuated MMP-1-mediated collagen cleavage (Fig.5C). However, treatment of the decorin-coated collagen fibrils with GzmB significantly increased the amount of collagen degradation to levels observed without decorin. GzmB alone did not cleave collagen fibrils (data not shown). Addition of Compound 20 inhibited the proteolytic activity of GzmB and thus protected decorin from cleavage, and in turn protected the collagen fibrils from MMP-1 proteolysis (Fig.5C).

## Discussion

Among all the organs, the study of skin is of particular importance because it is crucial to maintaining homeostasis in the body and has a strong societal impact due to its visibility, and it represents an ideal model organ for aging research (Wenk *et al*., 2001; Gilchrest & Krutmann, 2006). In contrast to intrinsically aged skin, which shows an overall reduction in cell numbers, UV-photoaged skin is characterized by epidermal hyperplasia and an increase in the number of dermal fibroblasts, mast cells, macrophages, and T cells (Bosset *et al*., 2003; Iddamalgoda *et al*., 2008). This infiltrate is indicative of a chronic inflammatory phenotype. Mast cells have a major role in the development of skin inflammatory processes. Cell-to-cell communications between mast cells and fibroblasts, as well as evidence of mast cell degranulation at sites of chronic inflammation have been documented (Metz *et al*., 2007). We observed an increase in epidermal thickness and mast cells in UV-irradiated skin. Immunostaining for GzmB revealed a direct colocalization of GzmB with the mast cell infiltrate, which supports previous studies demonstrating that mouse and human mast cells produce GzmB *in vivo* and *in vitro* and release it upon activation (Pardo *et al*., 2007; Strik *et al*., 2007). In addition, we observed increased GzmB expression in the keratinocytes of the epidermis after UV irradiation, also in line with previous reports (Hernandez-Pigeon *et al*., 2006, 2007). Aside from other possible inflammatory infiltrates expressing GzmB (e.g., cytotoxic lymphocytes), this represents a significant source of GzmB that may be released to the extracellular space and contribute to ECM degradation. Other studies comparing potential GzmB activity to neutrophil elastase in skin after UV irradiation have overlooked these important other sources (Li *et al*., 2013). Overall, GzmB is highly expressed after UV irradiation and may be a significant contributor to UV-induced ECM degradation, as well as in other pathologies characterized by chronic inflammation and ECM degradation.

Chronically irradiated skin is characterized by alterations in the dermal connective tissue. The ECM in the dermis mainly consists of collagen, elastin, proteoglycans (e.g., decorin), and fibronectin. In particular, collagen fibrils are important for the strength and resilience of skin, and alterations in their number and structure are thought to be responsible for wrinkle formation (Gilchrest & Krutmann, 2006). In the present study, using a mouse model of chronic UV irradiation over 20 weeks, GzmB deficiency significantly reduced the formation of deep coarse wrinkles and protected against the loss of collagen density in UV-treated skin. The natural bifringent properties of collagen were exploited to analyze collagen structure, organization, and density in unfixed, unstained, and thick skin samples in three-dimensional space using multiphoton microscopy. Highly ordered fibril-forming collagens produce second-harmonic generation (SHG) signals without the need for any exogenous label or processing, which correlate with the density and organization of the collagen matrix (Palero *et al*., 2007; Abraham *et al*., 2010). We suggest the results obtained using this technique are more robust and provide a better representation of the collagen density in the associated groups than picrosirius red staining, as while the analysis of fixed, thin-sliced sections may provide information in regard to content and morphological structure, important 3-dimensional and organizational features and properties may be overlooked or changed during processing. In addition, while we hypothesize that GzmB may be contributing to ECM degradation and result in a loss of collagen density, it is unlikely that a deficiency or inhibition of GzmB would be totally protective (as suggested by the picrosirius red staining). Other well-characterized processes, such as MMP induction and direct collagen degradation, may still occur, and the results obtained with multiphoton and SHG adequately reflect this physiology. Overall, our results provide evidence that GzmB deficiency protects against the loss of collagen density and the formation of deep wrinkles after UV irradiation, suggesting that GzmB contributes to collagen degradation and the appearance of photoaged skin.

As GzmB does not directly cleave collagen, we explored mechanisms to rationalize the differences in collagen density between GzmB-KO and wild-type mice. GzmB cleaves fibronectin (Buzza *et al*., 2005), and there was a significant difference in the amount of full-length fibronectin (FN) in skin homogenates of UV-treated skin. The quantification of a greater amount of full-length FN in GzmB-KO mouse skin is assumed to be due to the lack of cleavage by GzmB. Unfortunately, a limitation in the current study is in the ability to detect fibronectin fragments *in vivo*, as data showing a reduction in fibronectin fragments in GzmB-KO UV-irradiated skin would support this assumption. However, due to the relative protein load of a total skin homogenate, it is difficult to observe these fragments on Western blot. However, to further support our findings, GzmB treatment of mouse fibroblasts in culture resulted in a dose-dependent increase in FN fragmentation from the cell-derived matrix. FN is an important multifunctional ECM protein, containing an RGD integrin-binding site that facilitates cell binding and migration (Romberger, 1997). Previous studies suggest that GzmB cleaves FN just after the RGD-binding domain, implying that GzmB-mediated FN proteolysis may alter cell–matrix interactions (Buzza *et al*., 2005). In the present study, we observed a dose-dependent increase in cell rounding and detachment (after washing) following GzmB treatment that was accompanied by an increase in free FN fragments in the supernatant. FN fragments have been identified in the bodily fluids of many chronic inflammatory diseases, and it has been shown that exposure to certain FN fragments can significantly alter cell behavior, triggering different events from those elicited by binding of native fibronectin (Werb *et al*., 1989; Stanley *et al*., 2008). Of particular relevance, it is known that FN fragments can increase the expression of MMPs in fibroblasts (Werb *et al*., 1989; Huhtala *et al*., 1995; Tremble *et al*., 1995; Kapila *et al*., 1996). These studies have shown that rabbit synovial fibroblasts express basal levels of MMP-1 and MMP-3 when plated on intact FN, but elevated levels when plated on FN fragments containing the RGD cell-binding domain. They suggest the particular fragment modulates the expression of MMP-1 via its binding with the α5β1 integrin receptor, inducing a signaling cascade involving the AP-1 and PEA-3 elements on the MMP-1 promoter (AP-1 is also common to the MMP-3 promoter) (Tremble *et al*., 1995). Meanwhile, intact FN allows for cooperative signaling with multiple integrins outside this domain, which suppresses the induction (Huhtala *et al*., 1995). In our study, direct GzmB-mediated FN fragmentation increased the expression and release of MMP-1 and MMP-3 from human dermal fibroblasts. An increase in MMP-1 staining was also observed in skin from wild-type UV-irradiated mice. While it is possible that the induction of MMPs occurs through similar signaling pathways, further work is needed to support this hypothesis. Overall, while the induction of MMPs and the cleavage of collagen are well described in UV-irradiated skin (Gilchrest & Krutmann, 2006), GzmB may further mediate the expression through FN fragmentation to result in more collagen degradation as observed in our model.

There have been many studies exploring the role of topical tretinoin (retinoic acid) administration to improve the appearance of photoaged skin, which is reported to inhibit the induction of MMP-1, MMP-3, and MMP-9 in both the epidermis and dermis of skin exposed to UV irradiation (Griffiths *et al*., 1992; Fisher *et al*., 1997). Although they have shown promise in treatment of skin aging, irritant reactions such as burning, scaling, or dermatitis associated with retinoid therapy limit their acceptance by patients (Mukherjee *et al*., 2006). In addition, it is now recognized that MMPs are essential for the regulation of many normal physiological processes and that broad inhibition of MMPs may promote inflammation by suppressing MMP-mediated chemokine regulation (Murphy & Nagase, 2008; Dufour & Overall, 2013). In fact, it is now believed that 10 of the 24 MMPs have anti-inflammatory or antitumorigenic roles and have been termed drug ‘antitargets,’ meaning that the beneficial function of these enzymes should not be inhibited (Dufour & Overall, 2013). It follows that extracellular GzmB may be a beneficial target, as GzmB inhibition may reduce direct ECM cleavage (fibronectin), and the indirect induction of collagen-degrading MMPs to reduce ECM degradation after UV exposure.

In addition to the degradation of existing collagen through induction of MMPs, failure to replace damaged collagen is also thought to contribute to aging skin. Accordingly, in chronically UV-irradiated skin, collagen synthesis is down-regulated as compared to sun-protected skin (Fisher *et al*., 2000). It has been suggested that fibroblasts in severely UV-damaged skin have less interaction with intact collagen and are thus exposed to less mechanical tension, which is an important factor in collagen synthesis (Varani *et al*., 2004). It follows from our observations of GzmB-mediated MMP-1 expression and release from fibroblasts *in vitro* and a difference in collagen density *in vivo* that GzmB may promote collagen cleavage and degradation that, in turn, results in reduced collagen synthesis. Furthermore, it has been reported that collagen fragmentation promotes oxidative stress and elevates MMP-1 in fibroblasts in aged human skin (Fisher *et al*., 2009), resulting in a destructive cyclic mechanism that may be promoted by extracellular GzmB activity.

Decorin is a ubiquitous ECM protein that plays important roles in collagen fibrillogenesis and organization (Danielson *et al*., 1997). Binding of decorin to collagen fibrils protects collagen fibrils from MMP-mediated cleavage, with evidence suggesting that decorin binds in the same region at the MMP-1 cleavage site (Geng *et al*., 2006; Stuart *et al*., 2011). Decorin is cleaved by GzmB, and its degradation has been linked to collagen disorganization and disease progression (Boivin *et al*., 2012). There was a significant decrease in decorin staining in wild-type UV-irradiated mice, which was protected in GzmB-KO mice. Further, GzmB-mediated decorin cleavage rendered collagen fibrils more susceptible to MMP-1-mediated collagen degradation. This mechanism further supports the protective effect of GzmB deficiency observed *in vivo*.

**Figure 6:**
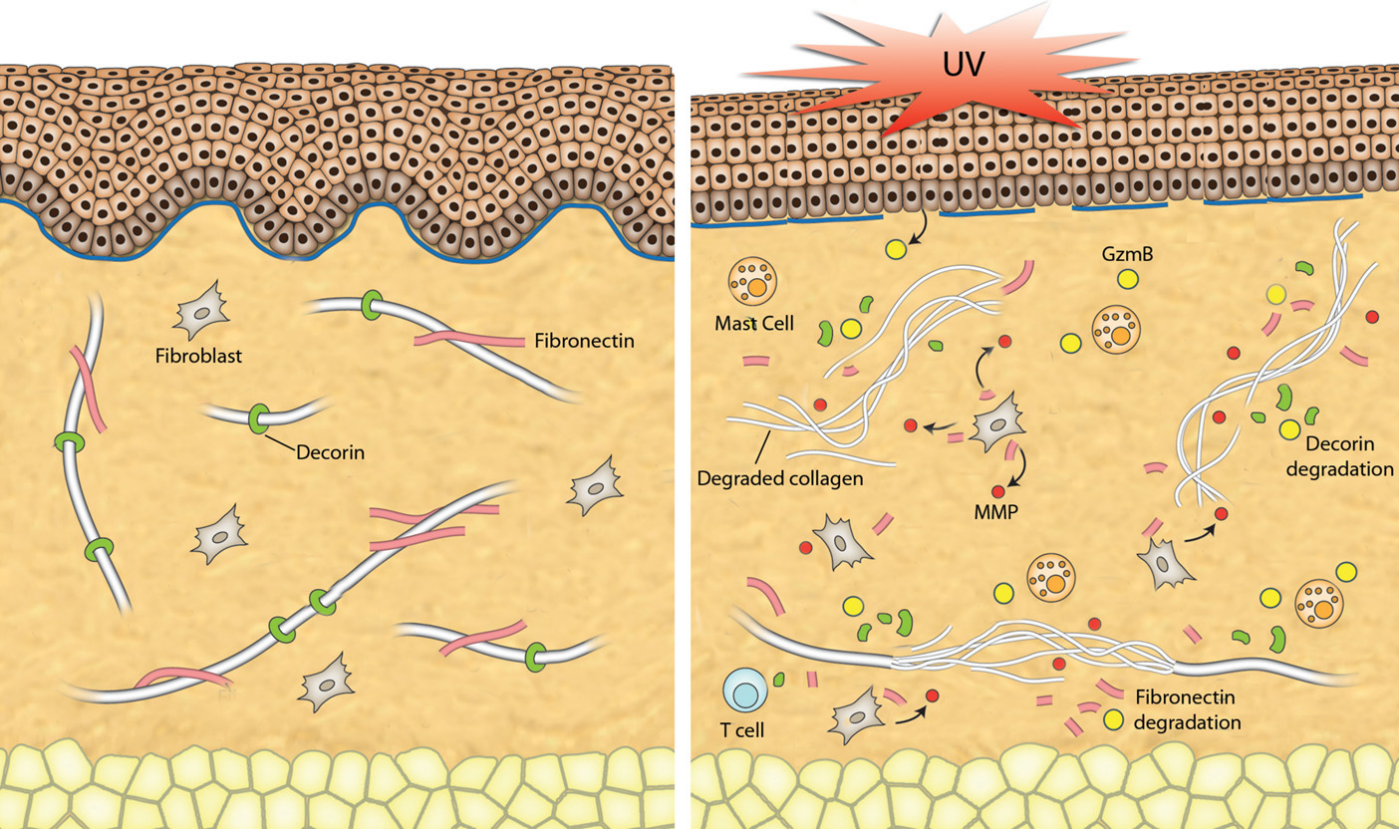
Summary of potential mechanisms of action for GzmB in UV-irradiated skin. After UV irradiation, GzmB is expressed and released into the extracellular space by a number of cells (keratinocytes, mast cells). GzmB can cleave fibronectin to form fragments that increase the expression and release of MMPs from fibroblasts. GzmB cleavage of decorin renders collagen fibrils more susceptible to MMP-mediated degradation. Overall, there is a loss of collagen density and organization leading to a phenotype of aged skin.

Overall, the present study suggests that GzmB contributes to both direct and indirect cleavage of ECM in skin after UV irradiation and promotes premature aging of the skin (summarized in Fig.6). UV irradiation induced GzmB expression in the skin and recruited GzmB-expressing immune cells such as mast cells. We propose a mechanism whereby UV-induced GzmB cleaves FN, producing FN fragments that augment MMP expression and secretion resulting in increased collagen degradation. GzmB-mediated decorin cleavage predisposes collagen fibrils to MMP-1 cleavage, further promoting a loss in collagen density. This research not only highlights the role GzmB in UV-induced damage of the skin, but also provides scope for implications in other age-related dermatological and nondermatological pathologies characterized by chronic inflammation, GzmB expression, and extracellular matrix degradation.

## Experimental procedures

### Mice

All animal procedures were performed in accordance with the guidelines for animal experimentation approved by the Animal Care Committee of the University of British Columbia (UBC). Female WT (C57BL/6J) and GzmB-KO (C57BL/6J background) mice were purchased from The Jackson Laboratory (Bar Harbor, ME, USA) and housed at the Genetic Engineered Models facility at the UBC Centre for Heart Lung Innovation, St Paul's Hospital, Vancouver, B.C. Animals were fed and watered *ad libitum* and maintained on a 12-h light/dark cycle. Different cohorts of mice were arranged as follows: WT control, killed at 7 weeks after study started (*n* = 10); wild-type control, killed at 20 weeks (*n* = 10); WT UV irradiated, killed after 7 weeks of exposure (*n* = 10); WT UV irradiated, 20 weeks (*n* = 10); GzmB-KO control, 20 weeks (*n* = 3); GzmB-KO UV irradiated, 7 weeks (*n* = 8); and GzmB-KO UV irradiated, 20 weeks (*n* = 8).

### UV irradiation

Solar-simulated UV (SSUV) radiation was produced by a planar array of eight fluorescent tubes comprising five UVA tubes (HOUVALITE F24T12/BL/HO; National Biological Corporation, Beachwood, Ohio, USA) interdispersed among three UVB tubes (FSX24T12/UVB/HO, National Biological Corporation), which were encased in a custom-modified Panosol II unit (Model UBB-247; National Biological Corporation). The tubes were allowed to stabilize for 15 min after switching on, and the temperature of the irradiation area was stably maintained using a fixed electric fan. This SSUV source provided 14.8 mW cm^-2^ UVA and 0.85 mW cm^-2^ UVB at the dorsal surface of the mice as measured by calibrated UVA (Model UVA-400C) and UVB (Model UVB-500C) meters (National Biological Corporation). The ratio of 17.5 of UVA to UVB is similar to that recorded from sunlight (Kollias *et al*., 2011).

Beginning at 6 weeks of age, the dorsal area of all mice was shaved with electric clippers once a week. Mice were exposed on the dorsum, unrestrained in a custom-designed wheel apparatus that slowly rotated under the irradiation source so as to provide a uniform dosage to each and every mouse. The minimal erythemal dose (MED) of SSUV was first established in a separate group of WT and GzmB-KO mice from a series of increasing exposures (time) and defined as the lowest dose resulting in a statistically significant increase in the mid-dorsal skinfold thickness at 48 h, measured with a spring micrometer (Mitutoyo, Model 700-118-20). While erythema is difficult to observe in the mouse, the MED can be quantified largely by its edema component or the acute inflammatory response to UV radiation which causes an increase in the dorsal skinfold thickness and is a useful equivalent to the minimal erythemal dose measurement in humans (Ibuki *et al*., 2007). The MED was determined by fixed increases in UVB dose to calculate exposure times to SSUV and found to be 70 mJ cm^-2^ UVB (1.22 J cm^-2^ UVA). There was no difference in the MED between genotypes (Fig. S4).

WT and GzmB-KO mice were then irradiated three times per week with exposure times calculated to provide a UVB dose equal to the UVB content of 1 × MED of SSUV for the first week (i.e., 70 mJ cm^-2^ UVB), 2 ×  MED of SSUV for the second week (i.e., 140 mJ cm^-2^ UVB), and 3 ×  MED of SSUV (i.e., 210 mJ cm^-2^ UVB) for the remaining 17 weeks (20 weeks total). Control animals were treated identically except that they were not irradiated.

### Clinical observations and scoring

Thickening, laxity, and wrinkling as well as erythemal reactions were evaluated weekly under isoflurane anesthesia. Digital photographs were captured at a fixed distance and angle from the dorsal area of both irradiated and control mice and later used to score each mouse on a 4-point scale of UV damage. A score of 0 represented the ‘normal’ appearance of a 6-week-old mouse entering the study. A score of 1 was attributed to the appearance of pigmented spots. A score of 2 was attributed to the appearance of pigmented spots and shallow wrinkles. Finally, a score of 3 represented the most severe phenotype observed, that of pigmented spots, and deep coarse wrinkles.

### Tissue collection, histology, and immunohistochemistry

Detailed information can be found in the Supporting Information section.

### Multiphoton microscopy

Multiphoton excitation was used to generate second-harmonic generation (SHG) signals from collagen as previously described (Abraham *et al*., 2010). For each sample, about 150–200 z-section of images were collected through decreasing tissue depths for a total thickness of approximately 130–180 μm per sample. Z-stacks were complied, and 3D image restoration was performed using Volocity software (Improvisions, Inc, Waltham, USA). SHG signals that fell within a set threshold were quantified for the entire 3D stack using Volocity software and normalized to the volume measured and expressed as collagen density.

### Skin homogenization and Western blot

Frozen skin samples (4 × 4 mm) were homogenized using a TissueLyser LT (Qiagen, Germantown, MD, USA) in ice-cold 7M Urea buffer containing protease inhibitor cocktail (Sigma-Aldrich, Saint Louis, MO, USA). Samples were homogenized, and sample buffer was exchanged to PBS using centrifuge filters (10 kDa, Millipore, USA). Total protein content was measured by Bradford Protein Assay, and samples were normalized to total protein for Western blots experiments. Samples were run on 10% polyacrylamide gels, transferred to nitrocellulose membranes, and probed for fibronectin (1:1000 dilution; Abcam, Cambridge, MA, USA) as previously described (Hendel & Granville, 2013). GAPDH was probed as a loading control. Antibody detection was achieved using the Odyssey Infrared Imaging System (LI-COR Biotechnology, Lincoln, NE, USA), and densitometry was performed with image j software.

### Fibroblast detachment assay

Detailed information can be found in the Supporting Information section.

### Fibronectin fragmentation and MMP expression

Wells of a 24-well culture plate were coated with 20 μg mL^-1^ fibronectin (FN) (Sigma) for 1 h at 37°C and then blocked with 1% sterile BSA. Wells were then treated with GzmB (100 nm) in serum/supplement-free Medium 106 (SFM; Cascade Biologics) for 2 h at 37°C. GzmB was inhibited by Compound 20 (50 μm) (Centre for Drug Research and Development, Vancouver, BC, USA) (Willoughby *et al*., 2002) for 30 min before primary human fibroblasts (HDFa, Life Technologies) were seeded (1.5 × 10^5^ cells/well in SFM) and incubated for 20 h at 37°C, 5% CO_2_. Control wells were prepared by incubating equivalent amounts of GzmB in SFM in a separate plate (previously blocked with 1% BSA) and then inhibiting with Compound 20. The GzmB/inhibitor media solution was transferred to wells that had been previously coated with FN and blocked as described above, before seeding of cells. After 20 h, supernatants were collected, protease inhibitor cocktail (Sigma) was added, and cell debris was removed by centrifugation. Supernatants were concentrated using centrifuge filters (10 kDa) so that the whole supernatant could be separated as a single sample on a 10% SDS-PAGE gel, transferred to nitrocellulose membranes, and probed for MMP-1 (1/1000, Abcam) or MMP-3 (1/1000, Abcam). Cell lysates from the same wells were collected and probed for GAPDH.

### Collagen degradation assay

Detailed information can be found in the Supporting Information section.

### Statistical analysis

One-way ANOVA with post hoc test, or *t*-test was used where appropriate for group comparison analyses with *P* < 0.05 considered significant. Statistical calculations were computed using graphpad prism version 5.01 for Windows, GraphPad software (San Diego, CA, USA).

## Supporting Information

**Fig. S1**Increase in epidermal and dermal thickness after UV irradiation. **Fig. S2** Quantification of immune cells (neutrophils, macrophages, T cells) in control and UV-irradiated mouse skin. **Fig. S3** GzmB-mediated FN fragments increase MMP-3 expression and release from fibroblasts. **Fig. S4** Minimal erythemal dose (MED) determinations in wild-type and GzmB-KO mice. **Data S1** Supplementary methods.
