# Supplementary material for: Granzyme B mediates both direct and indirect cleavage of extracellular matrix in skin after chronic low-dose ultraviolet light irradiation
Source: Aging Cell. 2014 Dec 11;14(1):67–77. doi: 10.1111/acel.12298 (PMC4326907; doi:10.1111/acel.12298)
Supplement: Supplementary file 1 [file acel0014-0067-sd1.pdf]

Supporting Information for:

Granzyme B mediates both direct and indirect cleavage of extracellular matrix in skin after chronic low-dose ultra-violet light irradiation

Leigh G. Parkinson[1,2], Ana Toro[1,2], Hongyan Zhao[1,2], Keddie Brown[1], Scott J. Tebbutt[1,3], David J. Granville[1,2*]

[1]*Centre for Heart Lung Innovation, St. Paul's Hospital, Vancouver, British Columbia, Canada*

[2]*Department of Pathology and Laboratory Medicine, University of British Columbia, Vancouver, British Columbia, Canada*

[3]*Department of Medicine, Division of Respiratory Medicine, University of British Columbia, Vancouver, British Columbia, Canada*

Supplementary Figure S1: Increase in epidermal and dermal thickness after UV irradiation.

Photoaged skin is characterized by epidermal hyperplasia, an increased deposition of glycosaminoglycans, accumulation of chondroitin/dermatan sulfates and increased cellularity, all of which can lead to an increase in skin thickness (Bernstein *et al.* 1996). An increase in skin thickness was also observed in a hairless mouse model of chronic UV irradiation (Moloney *et al.* 1992). Eventually over time, as observed humans, a decrease in skin thickness occurs as the matrix is remodeled (Gilchrest & Krutmann 2006).

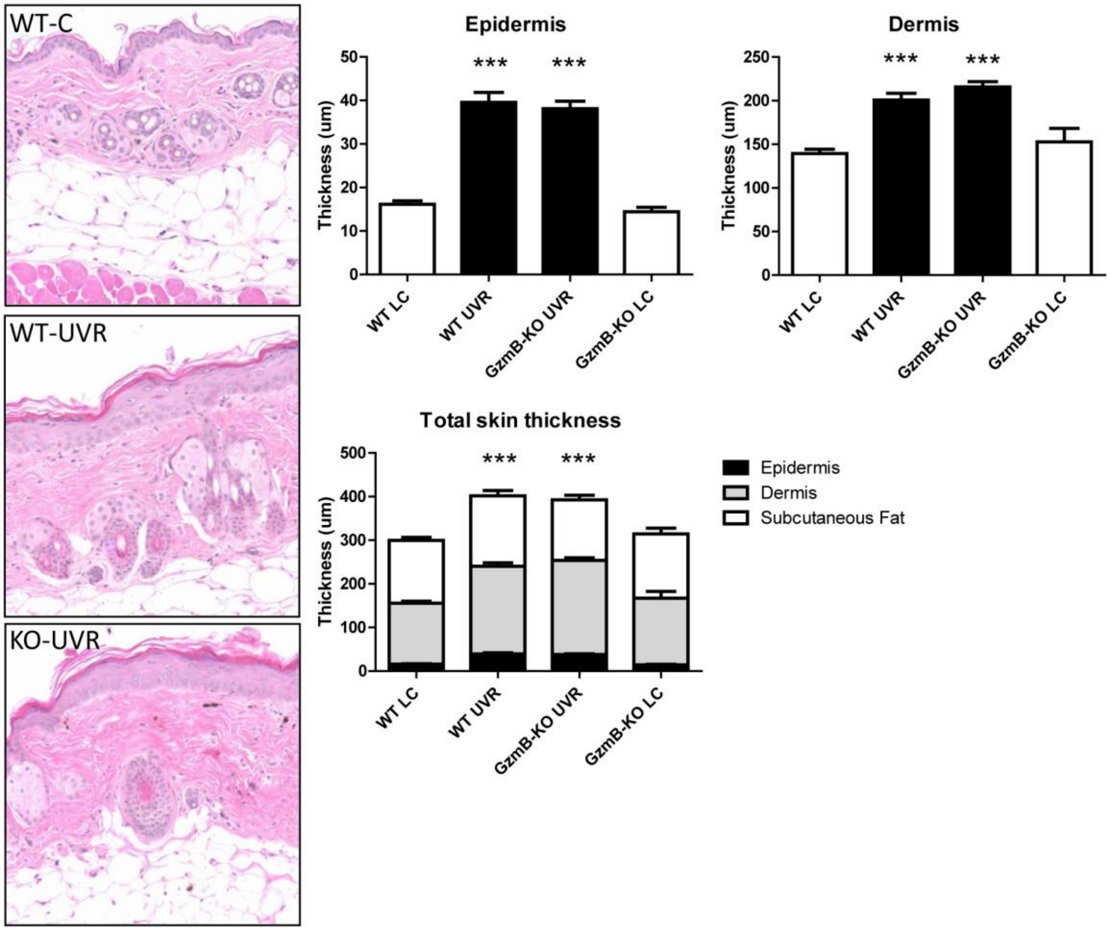

Fig S1: Increase in epidermal and dermal thickness after UV irradiation. Representative H&E stained sections of dorsal skin collected from wild-type non-irradiated control (WT-C), wild-type UV-irradiated (WT-UVR) and GzmB-KO UV-irradiated (KO-UVR) mice after 20 weeks. There was a significant increase in epidermal and dermal thickness after UV irradiation in both genotypes, leading to an overall increase in skin thickness (mean ± SEM; $p<0.05$ Tukey's multiple comparison).

Supplementary Figure S2: Quantification of immune cells (neutrophils, macrophages, T cells) in control and UV-irradiated mouse skin.

GzmB-deficiency did not affect the immune cell infiltrate to skin after UV-exposure.

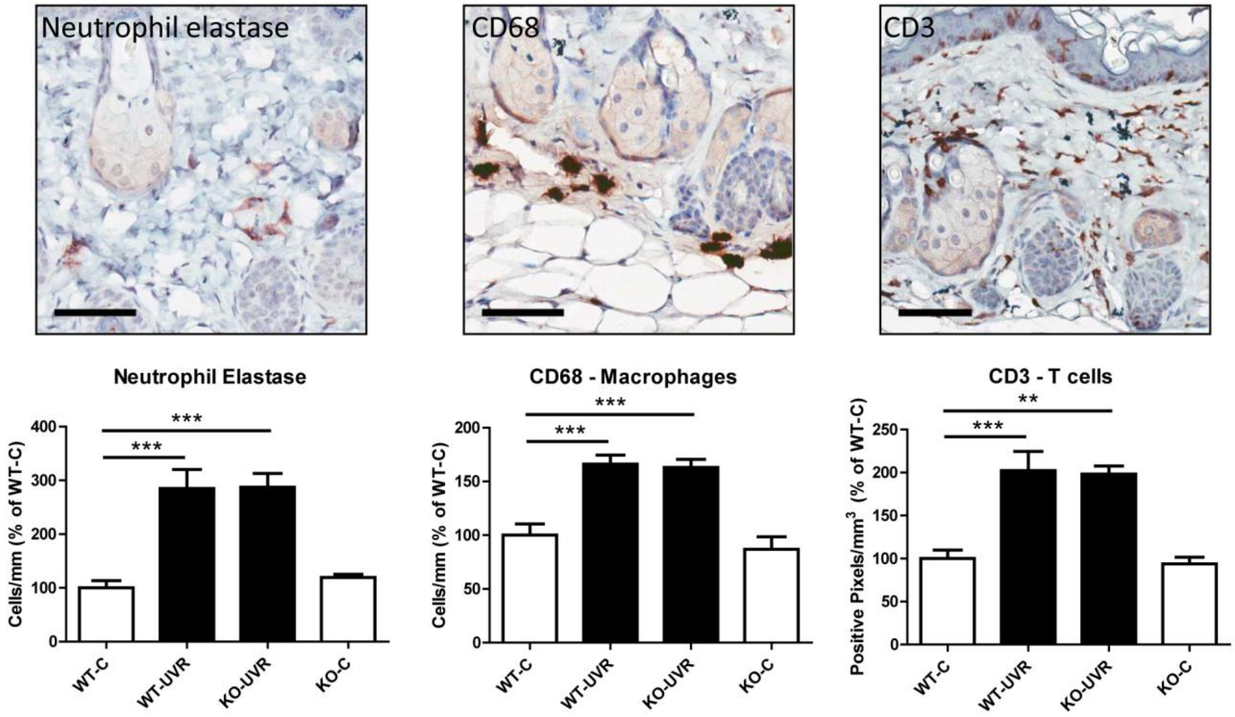

Fig S2: Quantification of immune cells in control and UV-irradiation mouse skin. Dorsal skin sections were immuno-stained for neutrophil elastase (neutrophils), CD68 (macrophages) and CD3 (T cells) and cells were either counted or the intensity of staining was measured via the number of positive pixels detected (above a set threshold) and normalized to area. Results are expressed at a percentage of wild-type non-irradiated control (WT-C) (mean ± SEM; *$p<0.05$, Tukey's multiple comparison). Scale bars = 60 μm.

Supplementary Figure S3: GzmB-mediated FN fragments increase MMP-3 expression and release from fibroblasts

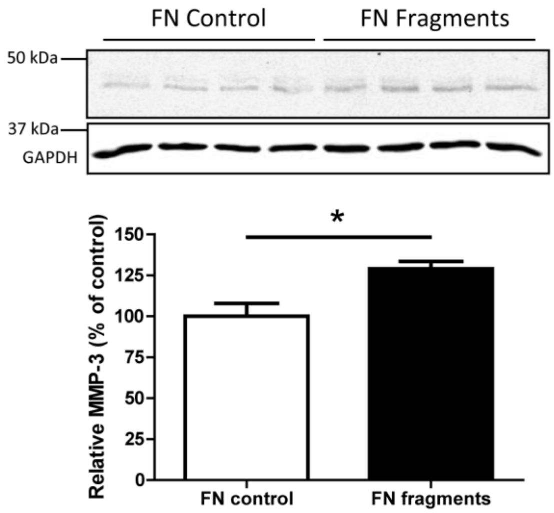

Figure S3: GzmB-mediated FN fragments increase MMP-3 expression and release from fibroblasts. Primary fibroblasts were added to GzmB-mediated FN-fragments and MMP-3 release was assayed in the supernatants after 20h by western blot. GAPDH was probed from cell lysates collected from the same wells as loading controls. Results are expressed as a percentage of intact fibronectin control (mean ± SEM from quadruplicate wells, *$p<0.05$ t-test).

Supplementary Figure S4: Minimal erythemal dose determinations (MED) in wild-type and GzmB-KO mice.

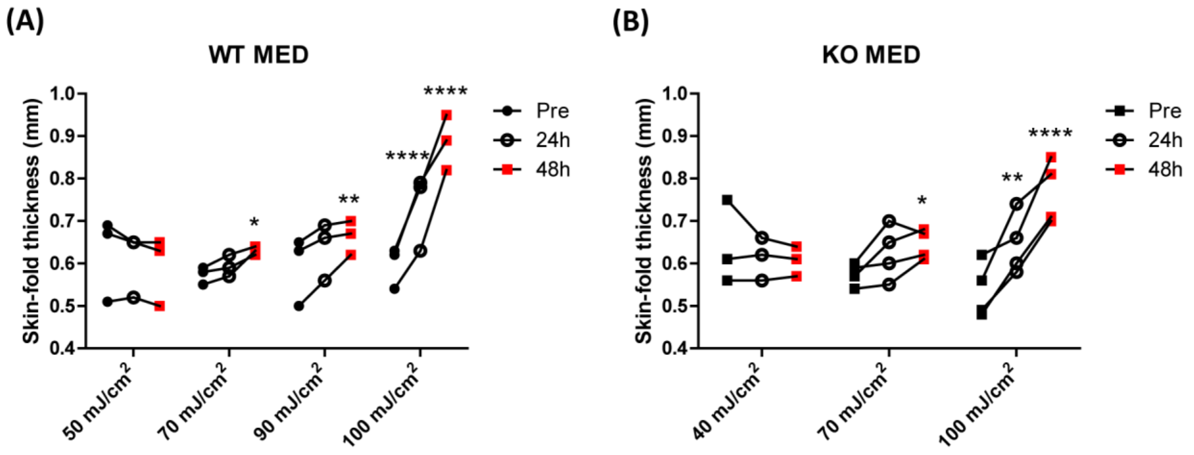

Fig S4: Minimal erythemal dose determinations (MED) in (A) wild-type and (B) GzmB-KO mice. MED was determined from a series of increasing exposures (time) and defined as the lowest dose resulting in a statistically significant increase in the mid-dorsal skin fold thickness at 48 h, measured with a spring micrometer. The MED was 70 mJ/cm^2 for both genotypes.

Supplementary Methods:

Tissue collection and processing

At 7 or 20 weeks, mice were euthanized by carbon dioxide inhalation and equivalent areas of dorsal skin were excised. An approximate 1x1cm piece of skin was fixed in 10% buffered formalin for 24 h and then processed to paraffin embedding for histological and immunohistochemical analyses. Skin samples were also flash frozen in liquid nitrogen and stored at -80 ^{o}C for subsequent multi-photon microscopy, protein extraction and western blot.

Histology and immunohistochemistry

Five micron cross-sections of skin were deparaffinized and stained with hematoxylin and eosin (H&E) for evaluation of morphology. Measurement of skin thickness was completed by scanning slides with an Aperio Scanscope and using the associated ImageScope software (Aperio Technologies Inc. v11.2.0.780) to take measurements across the entire section at multiple sites and averaged for each mouse. Collagen was observed in picrosirius red-stained sections using 100% polarized light and pictures were taken at a fixed exposure. Quantification of collagen was performed as previously described (Hiebert *et al.* 2013). Toluidine Blue (TBO) at pH 2.0 was used to stain mast cells.

GzmB (Abcam, ab4059), neutrophil elastase (Santa Cruz, SC-59338), CD68 (Abcam, ab125212), CD3 (Abcam, 5609), MMP-1 (Abcam, ab137332) and decorin (R&D, AF1060) immunohistochemistry was performed as previously described (Hiebert *et al.* 2011; Hiebert *et al.* 2013). The mast cell/GzmB co-stain was accomplished by first staining sections of tissue with TBO. After capturing images with the Aperio Scanscope system, the same sections were then immunostained for GzmB.

Mast cells, neutrophils and CD68 macrophages were manually counted and normalized to the length of tissue counted. CD3 T cells, MMP-1 and decorin staining was quantified using Aperio ImageScope. The number of color pixels (above a set threshold) were counted and expressed as the number of positive pixels per unit area. For all quantifications, at least three different areas were analyzed and averaged per mouse/section.

Fibroblast detachment assay

3T3 mouse fibroblasts were plated to wells of a 96-well tissue culture plate (4 x 10^4 cells/well) in DMEM containing 10% FBS and 1% P/S (Invitrogen), and allowed to adhere overnight. Cells were washed with PBS and incubated in serum free media (SFM) containing the indicated concentrations of GzmB (Beryllium, Seattle, WA) ± GzmB specific inhibitor Compound 20 (50 µM) (Centre for Drug Research and Development, Vancouver, BC) (Willoughby *et al.* 2002). Cells were incubated at 37°C for 7 h, after which images of the cells in the plate were captured under phase contrast using an Olympus microscope camera. In some experiments, the supernatant was collected, centrifuged to remove cellular debris and western blot was performed for fibronectin as previously described. Cells plated in parallel wells were lysed and probed for GAPDH on western blot as a loading control to show equivalent cell plating. For quantification of remaining adherent cells, MTS assays were performed after washing once with PBS to remove non-adherent cells, and results expressed as a percentage of untreated control.

Decorin cleavage assay

Decorin cleavage assay was performed as previously described (Hiebert *et al.* 2011). Decorin (0.4 ug, R&D Systems) was incubated with GzmB (100 nM) ± Inhibitor (Compound 20, 50 µM) for 8 hours at 37 ^{o}C. Samples were separated on a 10 % SDS-PAGE gel and proteins were visualized with SimplyBlue Safe Stain (Invitrogen).

Collagen degradation assay

Formation of collagen fibrils and decorin coating was based upon a previously described protocol, with modifications (Geng *et al.* 2006). For each sample, Type 1 collagen fibrils were formed in eppendorf tubes that had been blocked with 1% BSA and thoroughly washed. Briefly, 3.5 µL PureCol Collagen (Bovine Collagen, 3 mg/mL, Advanced BioMatrix, San Diego, CA) was mixed with 21.5 µL 10 mM HCl and 25 µL 50 mM Tris buffer (pH 7.5) and incubated for 2 h at 37°C, after which fibrils were recovered as a pellet by centrifugation. To coat the fibrils with decorin, fibril pellets were re-suspended in 33 µL Tris buffer, 15 µL recombinant decorin (3 µg, R&D Systems) and 2 µL 10 mM $ZnSO_4$ to facilitate binding. After incubating overnight at 37°C, the fibrils were recovered by centrifugation and then re-suspended in Tris buffer ± 100 nM

GzmB ± 50 µM Compound 20 for 8 h. Fibrils were again recovered by centrifugation. MMP-1 proenzyme (100 ng/sample) (Calbiochem, Millipore, San Deigo, CA) was activated by trypsin (50 nM) in MMP buffer (50 mM Tris, 0.5 M NaCl, 5 mM $CaCl_2$, pH 7.5) for l hour, after which trypsin activity was inhibited with soybean trypsin inhibitor. Fibrils were either re-suspended with activated MMP-1 or MMP buffer alone for 16 h at 37 °C. Samples were separated on 10 % SDS-PAGE gels and proteins were visualized in the gel with SimplyBlue Safe Stain (Invitrogen). Intact collagen was quantified via Image J.

Bernstein EF, Underhill CB, Hahn PJ, Brown DB, Uitto J (1996) Chronic sun exposure alters both the content and distribution of dermal glycosaminoglycans. *Br J Dermatol*. **135**, 255-262.

Geng Y, McQuillan D, Roughley PJ (2006) SLRP interaction can protect collagen fibrils from cleavage by collagenases. *Matrix Biol*. **25**, 484-491.

Gilchrest BA, Krutmann J (2006). *Skin Aging*. Berlin: Springer.

Hiebert PR, Boivin WA, Abraham T, Pazooki S, Zhao H, Granville DJ (2011) Granzyme B contributes to extracellular matrix remodeling and skin aging in apolipoprotein E knockout mice. *Exp Gerontol*. **46**, 489-499.

Hiebert PR, Wu D, Granville DJ (2013) Granzyme B degrades extracellular matrix and contributes to delayed wound closure in apolipoprotein E knockout mice. *Cell Death Differ*. **20**, 1404-1414.

Moloney SJ, Edmonds SH, Giddens LD, Learn DB (1992) The hairless mouse model of photoaging: evaluation of the relationship between dermal elastin, collagen, skin thickness and wrinkles. *Photochem Photobiol*. **56**, 505-511.

Willoughby CA, Bull HG, Garcia-Calvo M, Jiang J, Chapman KT, Thornberry NA (2002) Discovery of potent, selective human granzyme B inhibitors that inhibit CTL mediated apoptosis. *Bioorg Med Chem Lett*. **12**, 2197-2200.
